# A Computational Gene Expression Score for Predicting Immune Injury in Renal Allografts

**DOI:** 10.1371/journal.pone.0138133

**Published:** 2015-09-14

**Authors:** Tara K. Sigdel, Oriol Bestard, Tim Q. Tran, Szu-Chuan Hsieh, Silke Roedder, Izabella Damm, Flavio Vincenti, Minnie M. Sarwal

**Affiliations:** 1 Division of Transplant Surgery, Department of Surgery, University of California San Francisco, San Francisco, CA 94017, United States of America; 2 Kidney Transplant Unit, Bellvitge University Hospital, UB, Barcelona, Spain; Universidade de Sao Paulo, BRAZIL

## Abstract

**Background:**

Whole genome microarray meta-analyses of 1030 kidney, heart, lung and liver allograft biopsies identified a common immune response module (CRM) of 11 genes that define acute rejection (AR) across different engrafted tissues. We evaluated if the CRM genes can provide a molecular microscope to quantify graft injury in acute rejection (AR) and predict risk of progressive interstitial fibrosis and tubular atrophy (IFTA) in histologically normal kidney biopsies.

**Methods:**

Computational modeling was done on tissue qPCR based gene expression measurements for the 11 CRM genes in 146 independent renal allografts from 122 unique patients with AR (n = 54) and no-AR (n = 92). 24 demographically matched patients with no-AR had 6 and 24 month paired protocol biopsies; all had histologically normal 6 month biopsies, and 12 had evidence of progressive IFTA (pIFTA) on their 24 month biopsies. Results were correlated with demographic, clinical and pathology variables.

**Results:**

The 11 gene qPCR based tissue CRM score (tCRM) was significantly increased in AR (5.68 ± 0.91) when compared to STA (1.29 ± 0.28; p < 0.001) and pIFTA (7.94 ± 2.278 versus 2.28 ± 0.66; p = 0.04), with greatest significance for CXCL9 and CXCL10 in AR (p <0.001) and CD6 (p<0.01), CXCL9 (p<0.05), and LCK (p<0.01) in pIFTA. tCRM was a significant independent correlate of biopsy confirmed AR (p < 0.001; AUC of 0.900; 95% CI = 0.705–903). Gene expression modeling of 6 month biopsies across 7/11 genes (CD6, INPP5D, ISG20, NKG7, PSMB9, RUNX3, and TAP1) significantly (p = 0.037) predicted the development of pIFTA at 24 months.

**Conclusions:**

Genome-wide tissue gene expression data mining has supported the development of a tCRM-qPCR based assay for evaluating graft immune inflammation. The tCRM score quantifies injury in AR and stratifies patients at increased risk of future pIFTA prior to any perturbation of graft function or histology.

## Introduction

Kidney transplantation is the preferred modality for treatment of end-stage renal disease by any cause [1] and leads to better outcomes than dialysis [2]. However, long-term kidney allograft outcomes have not improved as expected despite a better understanding of the immune biology of allograft rejection and the advent of novel and more potent immunosuppressive agents [3]. Chronic allograft nephropathy continues to be the main reason for poor outcome and loss of graft and may be attributed to poor immune-risk assessment of transplant patients in current clinical practice. The main metrics used for monitoring a renal allograft are the relatively insensitive surrogate markers of allograft dysfunction such as serum creatinine [4, 5] as well as the use of allograft biopsies to directly diagnose histological lesions that are consistent with either acute rejection or interstitial fibrosis and tubular atrophy (IFTA). However, the serum creatinine increases due to many other reasons not related to allograft rejection such as immunosuppressive drug-related nephrotoxicity, urinary infections, or dehydration. The drift in serum creatinine is not predictive of tissue injury as the increase is seen late in injury, once allograft damage is already established; hence it has no utility for modifying treatment for prevention of rejection and/or IFTA. Furthermore, while the use of surveillance biopsies has been postulated as the gold standard tool for diagnosing allograft lesions, this approach is costly and invasive, even requiring sedation, particularly among pediatric transplant patients [6]. In addition, we and other have shown that immune injury predates chronic damage [7–10]. In a previously published paper we reported a common rejection module (CRM) consisting of 11 genes that were significantly overexpressed in acute rejection (AR) across all transplanted organs. The meta-analysis of eight independent transplant datasets from four organs yielded the CRM genes that could diagnose AR with high specificity and sensitivity in five additional independent cohorts [11]. In this study have analyzed the 11 CRM genes for their value as biomarker panel to diagnose AR and predict risk of accelerated or progressive IFTA injury (pIFTA). We sought out to validate the molecular changes within the allograft before and during acute rejection injury and evaluated if the combined expression of a finite set of the 11 CRM genes.

## Materials and Methods

### Study samples

All patients included in the study gave written informed consent to participate in the research, in full adherence to the Declaration of Helsinki. The study was approved by the institutional review board at Stanford University and University of California San Francisco. 146 renal allograft biopsies from 122 unique renal transplant patients were collected between 1 month– 10 years post-transplant as protocol biopsies or as indicated by acute graft dysfunction from pediatric and adult renal transplant patients with stable renal function (no-AR), AR, and pIFTA (for demographics see Table 1). Patients with acute rejection had biopsies collected prior to treatment intensification. Diagnosis of AR and IFTA was made by biopsy histology Banff classification [12]. All AR were T-cell mediated or mixed with T cell and antibody mediated injury (grade IA or higher) and pIFTA samples showed Banff scores grade II or higher (II and III), without showing any other specific accompanying lesions or AR. 1/3 of a needle biopsy core was collected in RNAlater solution and stored at -20°C until RNA extraction for the qPCR studies.

**Table 1:** Demographic Table.

Main demographics	AR(n = 54)	no-AR(n = 56)	pIFTA (n = 12)
Recipient gender (F, %)	20 (37)	25 (45)	5 (42)
Recipient age (years)	9.5±4.7	10.7±5.4	11.4±5.7
Donor gender (F, %)	26 (47)	20 (35)	6 (50)
Donor age (years)	27.9±13.6	30.0 ±11.5	33.5±6.8
Recipient Race (% Caucasion/Asian/Hispanic/African-American)	60/20/15/5	58/20/20/2	55/18/19/2
Type of Transplant (%)-Living	9 (17.3)	6 (10.5)	1 (8.3)
Type of Transplant (%)-Living-related	2 (3.8)	10 (17.5)	4(33.3)
Type of Transplant (%)-cadaver	7 (13.4)	19 (33.3)	2 (16.7)
*Cause of ESRD (%) (1/2/3/4/5/6)	10/5/8/9/6/16	14/5/5/8/8/16	2/0/3/2/2/3
Serum Creatinine (mg/dL) (mean+/- SD)	1.45±0.88	1.50±0.79	0.97± 0.0
post-transplantation (month)	33.99±31.58	14.00±9.15	11.32±9.91

*ESRD (1/2/3/4/5/6): 1, glomerulonephritis, 2, polycystic kidney disease, 3, renal dysplasia, 4, reflux nephropathy, 5, obstructive uropathy, 6 = other or unknown. None of these selected patients had delayed graft function. 20% of AR episodes were associated with documentation of non-adherence with medications (self-reported), appointments and/or laboratory measurements. Though the time post-transplant was significantly greater for AR (p<0.05); there was no difference in post-transplant time between no-AR and pIFTA.

### Patient demographics

This study used a total of 146 independent renal allografts collected from 122 unique patients with biopsy proven AR (n = 54) and no-AR (n = 92). 24 demographically matched patients with no-AR had 6 and 24 month paired protocol biopsies. Among these 24 patients all of them had histologically normal biopsies in 6 month post-transplantation time. However, only 12 of the 24 patients had evidence of progressive IFTA (pIFTA) on their 24 month biopsies. The remaining 12 patients were normal in their 24 month post-transplantation. The cross sectional samples were randomly split into two groups of 27 AR and 22 no-AR for the purpose to determine and validate a tCRM threshold for detection of AR. Of this latter group demographic variables were matched. For the 24 patients with paired samples everyone had normal kidney function and graft histology on the 6 month biopsies. Twelve patients in this group had pIFTA injury on their 24 month protocol biopsies (also labeled as progressors or P). The remaining 12 patients had histologically normal 24 month protocol biopsies (also called non-progressors or NP) (Table 1). All the patients received a calcineurin-inhibitor immunosuppressive regimen based on tacrolimus and induction therapy either with a T-cell depleting agent (Thymoglobulin) or with an anti-IL2 receptor monoclonal antibody (basiliximab). There was no statistical significance in between the demographical and the clinical parameters.

### Total RNA extraction, cDNA synthesis and qPCR

Total RNA was extracted from each biopsy using TRIzol Reagent (Invitrogen, Carlsbad, CA). RNA integrity was ensured using the RNA 6000 NanoLab Chip Kit (Agilent Technologies). cDNA synthesis was performed using 25 ng of extracted quality total RNA from the biopsy samples using SuperScript VILO Master Mix (Invitrogen, Carlsbad, CA) as per the manufacturer’s protocol. Briefly, specific target amplification was performed using 3.125 ng relative amount of cDNA using a pooled individual TaqMan real-time assays for the 11 genes investigated in multiplex with TaqMan PreAmp Master mix Life Technologies) to 10 μl final volume, for 18 cycles in a thermal cycler (Eppendorf Vapo-Protect, Hamburg, Germany), then diluted 1:20 with sterile water (Gibco, Invitrogen, Carlsbad, CA). The qPCR was performed on the QuantStudio 6 Flex System (Life Technologies) using 5μl of the diluted sample from the specific target amplification, along with the TaqMan Gene Expression Master Mix under standard conditions (2 min at 50°C, 10 min at 95°C, 40 cycles of 15s at 95°C, 1min at 60°C) using TaqMan gene expression assays (Life Technologies) for each of the 11 genes investigated: BASP1, CD6, CXCL10, CXCL9, INPP5D, ISG20, LCK, NKG7, PSMB9, RUNX3, TAP1. The relative amount of mRNA expression in each sample was calculated using the comparative threshold cycle (C_T_) method [13]. Ribosomal 18S RNA (18S) and Universal RNA (QIAGEN) were used for normalization of all genes since they showed the least variability in gene expression across all samples. Final gene expression results were converted to fold change. tCRM score was calculated by using the geometric mean of the fold changes of the respective genes.

### Data analyses

All data are presented as mean ± standard error of the mean (SEM). Groups were compared using the χ^2^ test for categorical variables, the two-way analysis of variance (ANOVA) or t-test for normally distributed data, and the nonparametric Kruskal–Wallis, Welch’s correction or Mann–Whitney U test for non-normally distributed variables. Bivariate correlation analyses were done using Pearson or Spearman tests for non-parametric variables. Sensitivity/specificity Receiver Operating Characteristic (ROC) curve analyses were performed to evaluate the most precise cut-off of the tissue CRM (tCRM) gene scores assessed at the time of biopsy, predicting the advent of acute rejection (AR). All predictive values were determined by calculating the area under the curve with SPSS software. Binary linear logistic regression analysis was performed to determine the independent correlation of several independent variables with the presence of AR. The statistical significance level was defined as p<0.05. Gene expression, tCRM scores and clinical variables were examined by multivariate analysis to determine predictive value. Cross sectional analysis was done to determine the significant increase in gene expression at event. Longitudinal studies were done to determine changes in gene expression over 2 years in patients with progressive IFTA (pIFTA).

## Results and Discussion

### The 11-gene intra-allograft common response module (tCRM) score accurately segregates acute rejection (AR) from stable (no-AR) kidney transplant patients in kidney tissue biopsies

**Fig 1:**
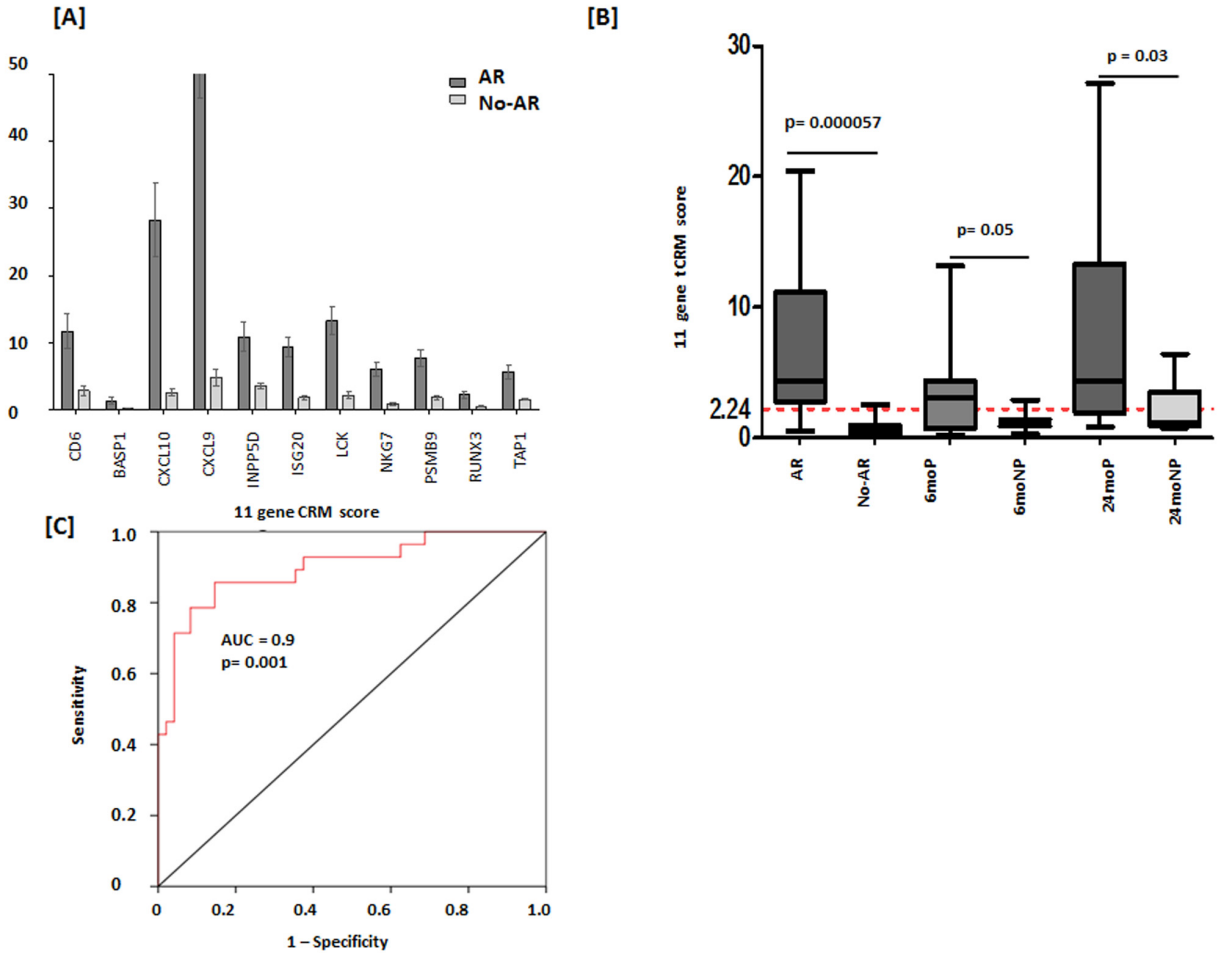
Gene expression of tCRM genes in AR. [A] Intra-allograft gene expression for tCRM genes increased in AR when compared to stable with the most significant differences seen between CXCL10 and CXCL9 (p = 0.0001). [B] The tCRM score is significantly increased in AR when compared to Stable (p = 0.0000057). Although there is increased in pIFTA progressors at 6 months, this difference did not meet significance (p = 0.05) until 24 months (p = 0.03). A threshold tCRM score of 2.24 as determined by the discovery set. [C] The tCRM score significantly predicts rejection with an AUC of 0.900 (p = 0.001, 95% CI = 0.823–0.976) with 82.7% sensitivity and 82.5% specificity.

The individual expressions of all 11 genes of the tCRM score showed a significant increase in AR, with the most significant increase seen in CXCL9 and CXCL10 (p = 0.0001 in both comparisons) between AR and no-AR (Fig 1). The tCRM score was also significantly increased in AR (mean ± SEM = 6.897 ± 1.082, n = 27) when compared to no-AR (mean ± SEM = 0.8144 ± 0.1374, n = 22, p = 0.000057) (Fig 1), and increased for pIFTA cohort for both the 6 and 24 month biopsies over the biopsies from patients who did not develop any histological changes (3.33 ± 1.00 versus 1.22 ± 0.2 at 6 months, p = 0.05; 7.94 ± 2.281 versus 2.28 ± 0.66 at 24 months; p = 0.03 at 24 months respectively) (Fig 1). Using the first set of 49 biopsies (27AR, 22no-AR), the tCRM score was found to be a significant correlate of Banff classified AR (AUC of 0.900 (p <0.001, 95% CI = 0.823–0.976) (Fig 1). With the aim of using the tCRM score as a binary variable to segregate patients at AR risk based on the tCRM score alone, the data was analyzed to choose a tCRM score of 2.24 which resulted in the greatest sensitivity and specificity for AR prediction (sensitivity = 82.7%, specificity = 82.5%) (Fig 1). When applied to the second independent biopsy set of 49 biopsies (27AR, 22no-AR) set, the tCRM threshold of 2.24 had a positive predictive value (PPV) for AR of 82.4%.

### The tCRM score correlates with the extent of AR lesions

**Fig 2:**
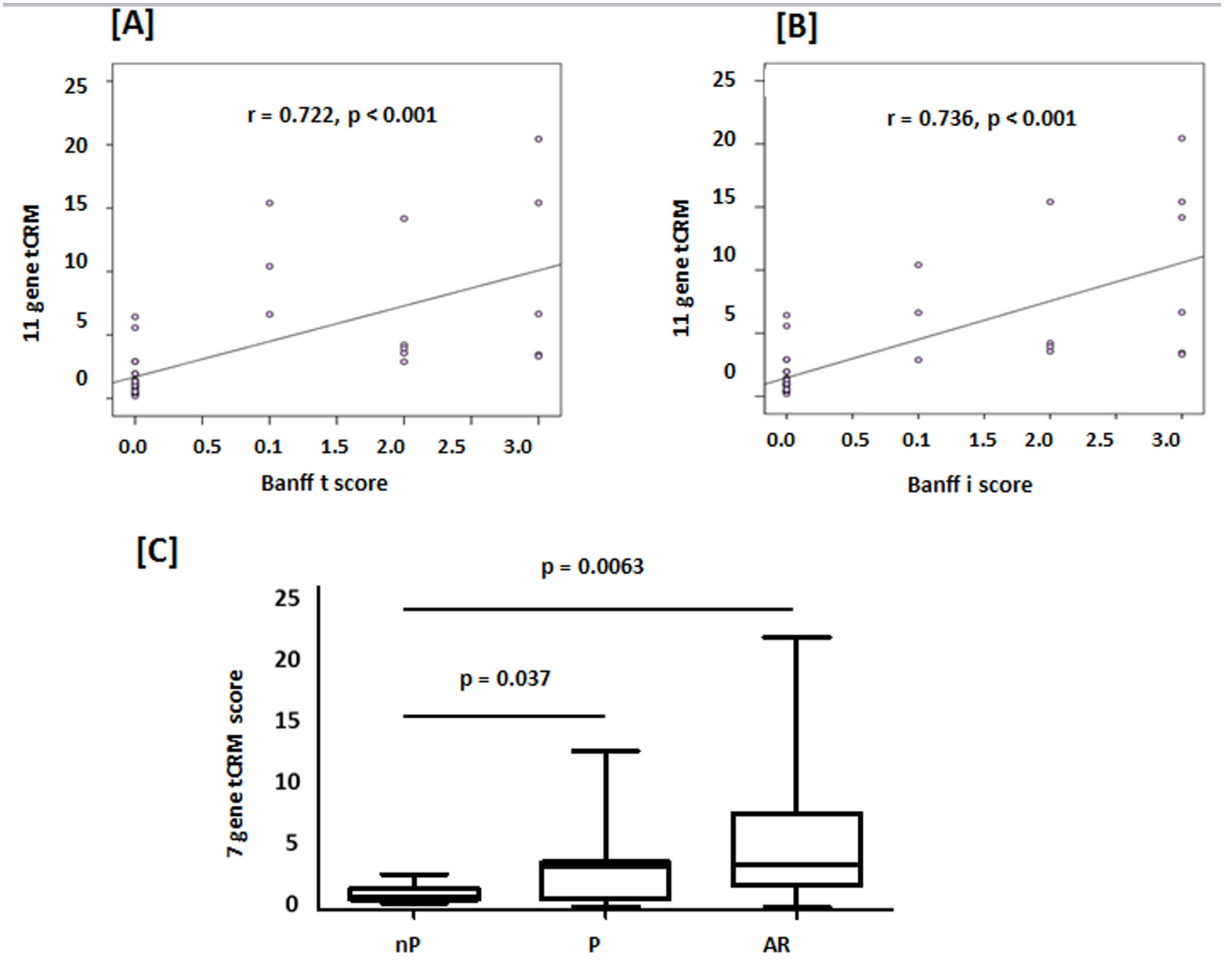
tCRM score correlates with AR lesions and chronic allograft nephropathy. [A] The tCRM score positively correlates significantly (p = 0.001) with the degree of infiltrates found on biopsy for the t-score = 0.722 and [B] i score = 0.736. [C] A tCRM score across a subset of 7 of the 11 genes differentiated most samples with pIFTA or progressors (3.29 ± 0.93) from no-AR patients (1.2 ± 0.18; p = 0.037). Stable/non-progressors (NP) and AR were highly distinguishable (1.198±0.1801 versus 5.582±0.8651; p = 0.0063). pIFTA/Progressors and AR were not different with regards to their tCRM scores (p = 0.16).

We evaluated whether the tCRM score was associated with the extent of the AR lesions observed in the matched biopsies. As observed in Fig 2, the tCRM score strongly correlated with the extent of the acute allograft lesions both at the tubular (Banff t score and the interstitum (Banff i score) kidney compartments (R = 0.72, p = 0.001 and R = 0.74, p = 0.001).

### The tCRM score is predictive of chronic allograft nephropathy

Biopsies from patients with chronic allograft changes as defined by the presence of IFTA were examined at 24 months, and in addition their biopsy pairs were also examined from their 6 month protocol biopsies. Significantly increased fold changes of all genes except BASP1 and CXLC10 were seen at both 6 and 24 months between pIFTA (P) and no-AR (NP) patients. While the tCRM score across all 11 genes was significant between pIFTA (P) and no-AR (NP) (p = 0.05), we found by using an adjusted R score analysis, that among the 11 genes, the greatest influence on pIFTA was from a subset of 7 genes (CD6, INPP5D, ISG20, NKG7, PSMB9, RUNX3, and TAP1), with maximal influence from CD6. A modified tCRM score across these 7 genes showed significantly greater scores in the 6 month biopsies of patients destined to develop pIFTA patients over time (p = 0.037) (Fig 2).

## Discussion

There is an urgent need in transplant medicine for developing reliable and non-invasive monitoring tools that may help transplant clinicians predict the risk of alloimmune-mediated allograft injury, preferentially before allograft damage has already been established. While a number of transcriptional biomarkers have been associated to AR [14–19], many studies either have limited sample sizes [20–23] or have focused on exploratory single biomarkers that may not necessarily reflect the crux of the molecular complexity in allograft rejection [24, 25]. Taking advantage of recently reported microarray meta-analyses of eight independent transplant datasets by combining effect size and p-values from our group [11], showing a common immune response module (CRM) of gene expression in allograft biopsies during AR, irrespective of the type of tissue organ, the main goal of this study was to validate these findings using the more practical method of PCR as well as determine a threshold score. We found that a qPCR-based intra-allograft tCRM score threshold of >2.24, can accurately distinguish AR from no-AR with high sensitivity and specificity. This data allows us to confirm the association of gene expression across this restricted set of genes with the histological immune injury of acute rejection, and also supports our earlier findings [7] that the molecular profile of immune injury is a threshold effect, with greater burden of injury in acute rejection, and similar, but lower injury burden in the subclinical injury of progressive IFTA. The panel of genes has differential impact on the injury phenotypes such as CXCL9 and CXCL10 are important layers in the injury of AR, and CD6 plays a major role in pIFTA, suggesting different roles for cell infiltration versus activation in acute and chronic graft injury. Cytokines are involved in all inflammatory responses. Being a class of small cytokines, the C-X-C motif chemokine ligand CXCL9 and CXCL10 play key roles in the initiation and development of acute transplant rejection [26]. Our observation of CXCL9 and CXCL10 being important factor in AR corroborates previously reported observations regarding their involvement in immune mediated graft injury [14–16, 19]. Our ongoing studies show the tCRM score is also elevated in BK viral nephritis in the graft (Sigdel *et al*, in submission), which supports data from previous reports of significant overlap in the molecular disturbances from the overlapping intragraft infiltrates in AR and BK viral nephritis [14, 27–30], though in the latter instance the diagnosis of BK viral infection is very evident also by BK viral urine and blood PCR positivity and tissue SV40 positive stains. Nevertheless, in histologically normal biopsies, and in the absence of AR and confounding intragraft infection, an elevated tCRM score is a strong harbinger of underlying molecular inflammation and can provide a very important warning to a clinician to closely monitor this patient for risk of pIFTA. Recent years have seen an increased effort to develop and validate new assays for early diagnosis of acute rejection of organ transplantation and transplant monitoring [31]. We and others have reported on potential surrogate gene biomarkers for acute rejection analyzing peripheral blood [32, 33], gene biomarkers using urine [34–36] and protein biomarkers analyzing serum and plasma [37], and urine [10, 38–41]; many of these are in varying stages of further validation. We suggest that the inclusion of the tCRM score into the analysis of the immune profile of protocol biopsies can be very valuable for risk analysis in the context of clinical trials, as the tCRM score may provide a companion diagnostic for differentiating patients into high and low immune risk, for stratification into different investigative treatment arms, with an increased margin for patient and graft safety. Because of the restricted sample size available for this study, we suggest that the value of the tCRM score to discriminate patients at increased risk of AR or IFTA, prior to any perturbation of graft function or histology may require further validation in a larger cohort of patients.

## Abbreviations

AR: acute rejection
no-AR: no acute rejection
IFTA: interstitial fibrosis tubular atrophy
pIFTA: progressive interstitial fibrosis tubular atrophy
P: progressors
NP: Non-progressors
CRM: common immune response module
tCRM: tissue common immune response module
qPCR: quantitative polymerase chain reaction
cDNA: complementary DNA
Ct: comparative threshold cycle
ROC: Receiver Operating Characteristic
ANOVA: analysis of variance
SEM: standard error of mean

## References

[1] Wolfe RA, Ashby VB, Milford EL, Ojo AO, Ettenger RE, Agodoa LY, et al Comparison of mortality in all patients on dialysis, patients on dialysis awaiting transplantation, and recipients of a first cadaveric transplant. The New England journal of medicine. 1999;341(23):1725–30. 10.1056/NEJM199912023412303 . 10580071

[2] Laupacis A, Keown P, Pus N, Krueger H, Ferguson B, Wong C, et al A study of the quality of life and cost-utility of renal transplantation. Kidney international. 1996;50(1):235–42. . 8807593 10.1038/ki.1996.307

[3] Lodhi SA, Lamb KE, Meier-Kriesche HU. Improving long-term outcomes for transplant patients: making the case for long-term disease-specific and multidisciplinary research. American journal of transplantation: official journal of the American Society of Transplantation and the American Society of Transplant Surgeons. 2011;11(10):2264–5. 10.1111/j.1600-6143.2011.03713.x . 21957938

[4] Pape L, Offner G, Ehrich JH, de Boer J, Persijn GG. Renal allograft function in matched pediatric and adult recipient pairs of the same donor. Transplantation. 2004;77(8):1191–4. . 15114083 10.1097/01.tp.0000120099.92220.7a

[5] Provoost AP, Wolff ED, de Keijzer MH, Molenaar JC. Influence of the recipient's size upon renal function following kidney transplantation. An experimental and clinical investigation. Journal of pediatric surgery. 1984;19(1):63–7. . 6366181 10.1016/s0022-3468(84)80018-4

[6] Davis ID, Oehlenschlager W, O'Riordan MA, Avner ED. Pediatric renal biopsy: should this procedure be performed in an outpatient setting? Pediatric nephrology. 1998;12(2):96–100. . 9543363 10.1007/s004670050412

[7] Naesens M, Khatri P, Li L, Sigdel TK, Vitalone MJ, Chen R, et al Progressive histological damage in renal allografts is associated with expression of innate and adaptive immunity genes. Kidney international. 2011;80(12):1364–76. 10.1038/ki.2011.245 . 21881554 PMC4492284

[8] Roedder S, Sigdel T, Salomonis N, Hsieh S, Dai H, Bestard O, et al The kSORT assay to detect renal transplant patients at high risk for acute rejection: results of the multicenter AART study. PLoS medicine. 2014;11(11):e1001759 10.1371/journal.pmed.1001759 25386950 PMC4227654

[9] Sigdel TK, Sarwal MM. Recent advances in biomarker discovery in solid organ transplant by proteomics. Expert review of proteomics. 2011;8(6):705–15. 10.1586/epr.11.66 22087656 PMC3282122

[10] Sigdel TK, Salomonis N, Nicora CD, Ryu S, He J, Dinh V, et al The identification of novel potential injury mechanisms and candidate biomarkers in renal allograft rejection by quantitative proteomics. Molecular & cellular proteomics: MCP. 2014;13(2):621–31. 10.1074/mcp.M113.030577 24335474 PMC3916658

[11] Khatri P, Roedder S, Kimura N, De Vusser K, Morgan AA, Gong Y, et al A common rejection module (CRM) for acute rejection across multiple organs identifies novel therapeutics for organ transplantation. The Journal of experimental medicine. 2013;210(11):2205–21. 10.1084/jem.20122709 24127489 PMC3804941

[12] Sis B, Mengel M, Haas M, Colvin RB, Halloran PF, Racusen LC, et al Banff '09 meeting report: antibody mediated graft deterioration and implementation of Banff working groups. American journal of transplantation: official journal of the American Society of Transplantation and the American Society of Transplant Surgeons. 2010;10(3):464–71. 10.1111/j.1600-6143.2009.02987.x . 20121738

[13] Livak KJ, Schmittgen TD. Analysis of relative gene expression data using real-time quantitative PCR and the 2(-Delta Delta C(T)) Method. Methods. 2001;25(4):402–8. 10.1006/meth.2001.1262 . 11846609

[14] Jackson JA, Kim EJ, Begley B, Cheeseman J, Harden T, Perez SD, et al Urinary chemokines CXCL9 and CXCL10 are noninvasive markers of renal allograft rejection and BK viral infection. American journal of transplantation: official journal of the American Society of Transplantation and the American Society of Transplant Surgeons. 2011;11(10):2228–34. 10.1111/j.1600-6143.2011.03680.x 21812928 PMC3184377

[15] Hu H, Kwun J, Aizenstein BD, Knechtle SJ. Noninvasive detection of acute and chronic injuries in human renal transplant by elevation of multiple cytokines/chemokines in urine. Transplantation. 2009;87(12):1814–20. . 19543058 10.1097/TP.0b013e3181a66b3e

[16] Schaub S, Nickerson P, Rush D, Mayr M, Hess C, Golian M, et al Urinary CXCL9 and CXCL10 levels correlate with the extent of subclinical tubulitis. American journal of transplantation: official journal of the American Society of Transplantation and the American Society of Transplant Surgeons. 2009;9(6):1347–53. 10.1111/j.1600-6143.2009.02645.x . 19459809

[17] Tatapudi RR, Muthukumar T, Dadhania D, Ding R, Li B, Sharma VK, et al Noninvasive detection of renal allograft inflammation by measurements of mRNA for IP-10 and CXCR3 in urine. Kidney international. 2004;65(6):2390–7. 10.1111/j.1523-1755.2004.00663.x . 15149352

[18] Segerer S, Cui Y, Eitner F, Goodpaster T, Hudkins KL, Mack M, et al Expression of chemokines and chemokine receptors during human renal transplant rejection. American journal of kidney diseases: the official journal of the National Kidney Foundation. 2001;37(3):518–31. . 11228176

[19] Hauser IA, Spiegler S, Kiss E, Gauer S, Sichler O, Scheuermann EH, et al Prediction of acute renal allograft rejection by urinary monokine induced by IFN-gamma (MIG). Journal of the American Society of Nephrology: JASN. 2005;16(6):1849–58. 10.1681/ASN.2004100836 . 15857922

[20] Shi S, Blumenthal A, Hickey CM, Gandotra S, Levy D, Ehrt S. Expression of many immunologically important genes in Mycobacterium tuberculosis-infected macrophages is independent of both TLR2 and TLR4 but dependent on IFN-alphabeta receptor and STAT1. Journal of immunology. 2005;175(5):3318–28. . 16116224 10.4049/jimmunol.175.5.3318

[21] Robertson G, Hirst M, Bainbridge M, Bilenky M, Zhao Y, Zeng T, et al Genome-wide profiles of STAT1 DNA association using chromatin immunoprecipitation and massively parallel sequencing. Nature methods. 2007;4(8):651–7. 10.1038/nmeth1068 . 17558387

[22] Kuznetsov VA, Orlov YL, Wei CL, Ruan Y. Computational analysis and modeling of genome-scale avidity distribution of transcription factor binding sites in chip-pet experiments. Genome informatics International Conference on Genome Informatics. 2007;19:83–94. . 18546507

[23] Ellis SL, Gysbers V, Manders PM, Li W, Hofer MJ, Muller M, et al The cell-specific induction of CXC chemokine ligand 9 mediated by IFN-gamma in microglia of the central nervous system is determined by the myeloid transcription factor PU.1. Journal of immunology. 2010;185(3):1864–77. 10.4049/jimmunol.1000900 20585034 PMC2925661

[24] Sarwal M, Chua MS, Kambham N, Hsieh SC, Satterwhite T, Masek M, et al Molecular heterogeneity in acute renal allograft rejection identified by DNA microarray profiling. The New England journal of medicine. 2003;349(2):125–38. 10.1056/NEJMoa035588 . 12853585

[25] Der SD, Zhou A, Williams BR, Silverman RH. Identification of genes differentially regulated by interferon alpha, beta, or gamma using oligonucleotide arrays. Proceedings of the National Academy of Sciences of the United States of America. 1998;95(26):15623–8. 9861020 10.1073/pnas.95.26.15623 PMC28094

[26] Zhuang J, Shan Z, Ma T, Li C, Qiu S, Zhou X, et al CXCL9 and CXCL10 accelerate acute transplant rejection mediated by alloreactive memory T cells in a mouse retransplantation model. Experimental and therapeutic medicine. 2014;8(1):237–42. 10.3892/etm.2014.1714 24944628 PMC4061216

[27] Mannon RB, Hoffmann SC, Kampen RL, Cheng OC, Kleiner DE, Ryschkewitsch C, et al Molecular evaluation of BK polyomavirus nephropathy. American journal of transplantation: official journal of the American Society of Transplantation and the American Society of Transplant Surgeons. 2005;5(12):2883–93. 10.1111/j.1600-6143.2005.01096.x . 16303001

[28] Girmanova E, Brabcova I, Klema J, Hribova P, Wohlfartova M, Skibova J, et al Molecular networks involved in the immune control of BK polyomavirus. Clinical & developmental immunology. 2012;2012:972102 10.1155/2012/972102 23251224 PMC3521483

[29] Dadhania D, Snopkowski C, Ding R, Muthukumar T, Lee J, Bang H, et al Validation of noninvasive diagnosis of BK virus nephropathy and identification of prognostic biomarkers. Transplantation. 2010;90(2):189–97. 20526237 10.1097/TP.0b013e3181e2a932 PMC2989149

[30] Lubetzky M, Bao Y, P OB, Marfo K, Ajaimy M, Aljanabi A, et al Genomics of BK viremia in kidney transplant recipients. Transplantation. 2014;97(4):451–6. . 24310299 10.1097/01.TP.0000437432.35227.3e

[31] Lo DJ, Kaplan B, Kirk AD. Biomarkers for kidney transplant rejection. Nature reviews Nephrology. 2014;10(4):215–25. 10.1038/nrneph.2013.281 . 24445740

[32] Li L, Khatri P, Sigdel TK, Tran T, Ying L, Vitalone MJ, et al A peripheral blood diagnostic test for acute rejection in renal transplantation. American journal of transplantation: official journal of the American Society of Transplantation and the American Society of Transplant Surgeons. 2012;12(10):2710–8. 10.1111/j.1600-6143.2012.04253.x 23009139 PMC4148014

[33] Roedder S, Sigdel T, Salomonis N, Hsieh S, Dai H, Bestard O, et al The kSORT assay to detect renal transplant patients at high risk for acute rejection: results of the multicenter AART study. PLoS medicine. 2014;11(11):e1001759 10.1371/journal.pmed.1001759 25386950 PMC4227654

[34] Fairchild RL, Suthanthiran M. Urine CXCL10/IP-10 Fingers Ongoing Antibody-Mediated Kidney Graft Rejection. Journal of the American Society of Nephrology: JASN. 2015 10.1681/ASN.2015040353 . 25948874 PMC4625686

[35] Muthukumar T, Lee JR, Dadhania DM, Ding R, Sharma VK, Schwartz JE, et al Allograft rejection and tubulointerstitial fibrosis in human kidney allografts: interrogation by urinary cell mRNA profiling. Transplantation reviews. 2014;28(3):145–54. 10.1016/j.trre.2014.05.003 24929703 PMC4118424

[36] Lee JR, Muthukumar T, Dadhania D, Ding R, Sharma VK, Schwartz JE, et al Urinary cell mRNA profiles predictive of human kidney allograft status. Immunological reviews. 2014;258(1):218–40. 10.1111/imr.12159 24517436 PMC3947569

[37] Chen R, Sigdel TK, Li L, Kambham N, Dudley JT, Hsieh SC, et al Differentially expressed RNA from public microarray data identifies serum protein biomarkers for cross-organ transplant rejection and other conditions. PLoS computational biology. 2010;6(9). 10.1371/journal.pcbi.1000940 20885780 PMC2944782

[38] Schaub S, Rush D, Wilkins J, Gibson IW, Weiler T, Sangster K, et al Proteomic-based detection of urine proteins associated with acute renal allograft rejection. Journal of the American Society of Nephrology: JASN. 2004;15(1):219–27. . 14694176 10.1097/01.asn.0000101031.52826.be

[39] Sigdel TK, Kaushal A, Gritsenko M, Norbeck AD, Qian WJ, Xiao W, et al Shotgun proteomics identifies proteins specific for acute renal transplant rejection. Proteomics Clinical applications. 2010;4(1):32–47. 10.1002/prca.200900124 20543976 PMC2883247

[40] Ling XB, Sigdel TK, Lau K, Ying L, Lau I, Schilling J, et al Integrative urinary peptidomics in renal transplantation identifies biomarkers for acute rejection. Journal of the American Society of Nephrology: JASN. 2010;21(4):646–53. 10.1681/ASN.2009080876 20150539 PMC2844301

[41] Sigdel TK, Ng YW, Lee S, Nicora CD, Qian WJ, Smith RD, et al Perturbations in the urinary exosome in transplant rejection. Frontiers in medicine. 2014;1:57 10.3389/fmed.2014.00057 25593928 PMC4292055

